# Physical and Functional Properties of Sweet Potato Flour: Influence of Variety and Drying Method

**DOI:** 10.3390/molecules30081846

**Published:** 2025-04-20

**Authors:** Nelson Pereira, Ana Cristina Ramos, Marco Alves, Vítor D. Alves, Margarida Moldão, Marta Abreu

**Affiliations:** 1 Unidade de Tecnologia e Inovação, INIAV—Instituto Nacional de Investigação Agrária e Veterinária, 2780-157 Oeiras, Portugal; cristina.ramos@iniav.pt; 2 LEAF—Linking Landscape, Environment, Agriculture and Food Research Center, Instituto Superior de Agronomia, Universidade de Lisboa, 1349-017 Lisboa, Portugal; vitoralves@isa.utl.pt (V.D.A.); mmoldao@isa.utl.pt (M.M.); 3 GeoBioTec—Geobiociências, Geoengenharias e Geotecnologias, NOVA School of Science and Technology, Universidade Nova de Lisboa, 2829-516 Caparica, Portugal; 4 INOV.LINEA/TAGUSVALLEY—Science and Technology Park, 2200-062 Abrantes, Portugal; marco_alves@tagusvalley.pt; 5 Associate Laboratory TERRA, Instituto Superior de Agronomia, Universidade de Lisboa, 1349-017 Lisboa, Portugal

**Keywords:** hot-air drying, freeze-drying, flour properties, water absorption capacity, oil absorption capacity, gelation behaviour, gluten-free

## Abstract

Sweet potato (*Ipomoea batatas* (L.) Lam.; SP) flour enhances food nutrition and bioactivity while functioning as a thickening/gelling agent. This study investigated the impact of two drying methods [hot-air (75 °C/20 h) and freeze-drying (−41–30 °C/70 h)] on the physical–functional properties of flours from three SP varieties: *Bonita* (white-fleshed), *Bellevue* (orange-fleshed), and *NP1648* (purple-fleshed). Particle size, morphology, water/oil absorption capacities (WAC/OAC), bulk density, swelling power (SwP), water solubility (WS), foaming/emulsifying properties, least gelation concentration (LGC), and gelatinisation temperature (GT) were analysed. Both the drying method and variety significantly influenced these properties. Hot-air-dried flours exhibited bimodal particle distribution, compact microstructure, and aggregated starch granules, yielding higher WAC (≈3.2 g/g) and SwP (≈3.6 g/g). Freeze-dried flours displayed smaller particles, porous microstructure, and fragmented granules, enhancing OAC (≈3.0 g/g) and foaming capacity (≈17.6%). GT was mainly variety-dependent, increasing as *Bellevue* (74.3 °C) < *NP1648* (78.5 °C) < *Bonita* (82.8 °C), all exceeding commercial potato starch (68.7 °C). *NP1648* required lower LGC (10% vs. 16% for others). All flours exhibited high WS (24–39.5%) and emulsifying capacity (≈44%). These results underscore the importance of selecting the appropriate drying method and variety to optimise SP flour functionality for targeted food applications. Freeze-dried flours might suit aerated/oil-retentive products, while hot-air-dried flours could be ideal for moisture-sensitive formulations.

## 1. Introduction

Sweet potato (*Ipomoea batatas* (L.) Lam.) is a nutrient-dense root vegetable rich in starch, dietary fibre, minerals, and bioactive compounds, including carotenoids and phenolics such as anthocyanins [1]. Beyond its widespread consumption in fresh form, processing sweet potato (SP) into flour has emerged as a sustainable strategy to valorise post-harvest surpluses and expand its applications in food formulations. This valorisation not only enhances food security by promoting a stable and diversified supply but also improves the economic viability of SP production [2].

Sweet potato flour has garnered significant attention in the food industry due to its multifunctionality. It enhances food products’ nutritional profile, bioactivity, colour, and sweetness while imparting valuable functional properties (such as high water and oil absorption, swelling power, among others), gelation capacity, and thickening ability [3,4]. Gelation capacity is the minimum concentration required to form a three-dimensional network capable of retaining water, whereas thickening ability is the capacity to increase viscosity without necessarily forming a structured gel [5,6]. These attributes facilitate SP flour’s incorporation into various applications, including baked goods, snacks, soups, and sauces [4]. Given the growing consumer demand for health-promoting foods with enhanced sensory attributes, SP flour also represents a promising gluten-free alternative for innovative product development.

Rheological properties are essential analytical tools for understanding food ingredients’ structural organisation and functionality. During processing, starches and proteins can undergo structural modifications that may influence their functional behaviour, resulting in the formation of viscous dispersion, solutions, or gels, depending on temperature and concentration [7]. Understanding the interrelationships among rheological parameters is of interest in predicting ingredient functional performance. In SP flour, starch is the predominant macronutrient, and its variety-specific characteristics are crucial in determining its technological behaviour as a food ingredient. For instance, starch gelatinisation temperature is specific to each variety and depends on factors such as granule size, amylose-to-amylopectin ratio, and intra- and inter-molecular interactions, all of which can impact viscosity, stability, and texture in food formulations [8,9].

The quality and performance of SP flour are further modulated by the drying method employed and by the inherent variability among SP varieties [10]. Drying processes such as hot-air (HAD) and freeze-drying (FD) could differentially impact starch structure preservation, thereby altering essential functional properties, including gelatinisation temperature, least gelation concentration, and water and oil absorption capacities [8]. A study by Pereira et al. [11] evaluated the impact of different drying methods (hot-air and freeze-drying) on the bioactive quality of SP flour from diverse varieties, revealing that varietal differences played a more significant role in preserving the bioactive composition than the drying method itself. However, the effects of these factors on functional properties remain underexplored.

Based on the hypothesis that both the drying method and SP variety significantly influence the starch structure and, consequently, the flour’s rheological behaviour, this study aims to evaluate the several physical and functional properties of SP flours produced from three distinct varieties using two drying methods (hot-air drying and freeze-drying). This research provides insights into tailoring SP flour as a functional ingredient for diverse food formulations.

## 2. Results and Discussion

This section details the impact of both the drying method and the SP variety on the physical and functional properties of the flours.

### 2.1. Particle Size Distribution

**Figure 1:**
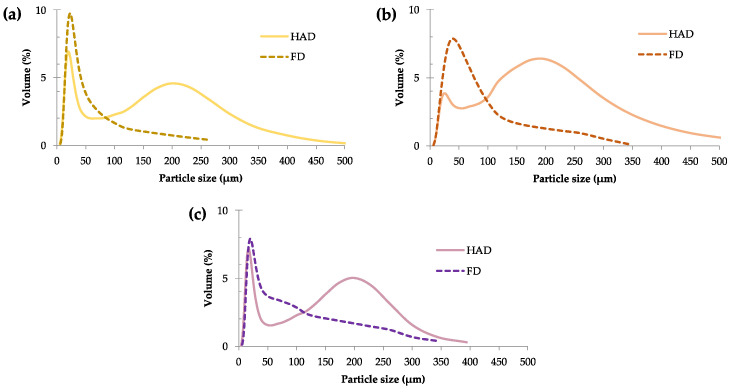
Particle size distribution curves of hot-air-dried (HAD) and freeze-dried (FD) flours from three SP varieties: (**a**) *Bonita*, (**b**) *Bellevue*, and (**c**) *NP1648*.

The particle size distribution of SP flours (Figure 1) was significantly influenced by both the SP variety (F = 3432) and drying method (F = 2076).

Across all varieties, freeze-dried flours exhibited a marked shift towards smaller particle sizes compared to hot-air-dried flours. Freeze-dried flours showed an unimodal distribution, with a dominant peak in the finer particle size range (≤50 µm). In contrast, hot-air-dried flours displayed broader, bimodal distributions, with peaks extending across a wider range of particle sizes, including both fine (≤50 µm) and larger particles (>200 µm). Similar results were reported by Buzera et al. [12], who found that freeze-dried potato flour had a smaller particle size (56.5 µm) compared to its hot-air-dried counterpart (307.5 µm). This difference was attributed to the sublimation process in FD, which removes water without structural collapse, producing brittle and porous particles more susceptible to fragmentation during milling, leading to finer particle sizes [13]. On the other hand, the thermal effects of HAD, such as induced partial gelatinisation or possible starch granule agglomeration, may contribute to the formation of coarser particles [14]. Furthermore, Buzera et al. [12] highlighted an unimodal particle size distribution for freeze-dried flours, while oven-dried flours displayed bimodal distributions.

The effect of variety was more pronounced in hot air-dried flours than in freeze-dried ones. Among hot-air-dried flours (Figure 1), *Bonita* and *NP1648* varieties exhibited a higher proportion of smaller particles [d(0.5) = 17.4 µm]. In comparison, *Bellevue* was characterised by a higher proportion of larger particles [d(0.5) = 185.9 µm], highlighting the intrinsic nature of each variety. Furthermore, the particle sizes evaluated in *NP1648* were consistent with the reported values [d(0.5) = 15.8 µm] for hot-air-dried SP flour in purple-fleshed varieties [15].

### 2.2. Morphology of Flour Particles

**Figure 2:**
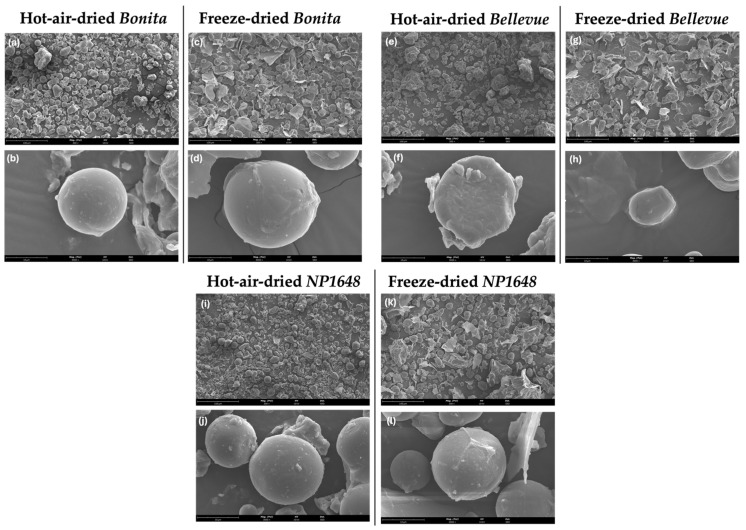
SEM micrographs of hot-air-dried and freeze-dried flours from three SP varieties (*Bonita*, *Bellevue*, and *NP1648*) at two magnitudes. (**a**) Hot-air-dried *Bonita* flour at 300×; (**b**) Hot-air-dried *Bonita* flour at 3000×; (**c**) Freeze-dried *Bonita* flour at 300×; (**d**) Freeze-dried *Bonita* flour at 3000×; (**e**) Hot-air-dried *Bellevue* flour at 300×; (**f**) Hot-air-dried *Bellevue* flour at 3000×; (**g**) Freeze-dried *Bellevue* flour at 300×; (**h**) Freeze-dried *Bellevue* flour at 3000×; (**i**) Hot-air-dried *NP1648* flour at 300×; (**j**) Hot-air-dried *NP1648* flour at 3000×; (**k**) Freeze-dried *NP1648* flour at 300×; (**l**) Freeze-dried *NP1648* flour at 3000×.

Figure 2 shows the SEM micrographs of the SP flours at two magnifications (300× and 3000×). The notable differences in particle surface morphology between the flours dried by the two drying methods underscore their influence on the physical properties of SP flours.

Across all varieties, hot-air-dried flours (Figure 2a,b,e,f,i,j) predominantly exhibited intact starch granules, retaining their spherical morphology. Some agglomeration and surface modification were observed, likely due to moisture loss and localised heat exposure, which may promote partial gelatinisation and granule restructuring. However, pronounced gelatinisation was not evident, as granules largely maintained their structural integrity [16]. The uniform and spherical particles appeared less aggregated, aligning with previous findings that HAD preserves granule distinctiveness [17]. In contrast, freeze-dried flours (Figure 2c,d,g,h,k,l) displayed a more fragmented and heterogeneous microstructure, characterised by disrupted starch granules, lamellar formations, and a porous matrix. The effect of freezing associated with freeze-drying exerts structural changes on the starch that can be attributed to the mechanical stress caused by ice crystal formation, disrupting its matrix, increasing brittleness and leading to the partial collapse of the granules [18,19]. The authors suggest that the minimal thermal exposure in FD preserves starch crystallinity while promoting physical disintegration.

Consistent with previous studies on SP purple pulp [15] and potato [12] flours, the starch granules generally retained their characteristic round shapes (Figure 2b,d,f,h,j,l), regardless of SP variety and drying method. However, the drying conditions significantly affected the microstructure. Similar trends were reported by Ahmed et al. [20], who observed that different pretreatments and drying methods resulted in notable variations in the microstructure of SP flour.

### 2.3. Bulk Density

The bulk density of SP flours (Table 1) was significantly influenced by SP variety (F = 132) rather than the drying method (F = 30).

Except for *NP1648*, the hot-air-dried flours had higher bulk density values than their freeze-dried counterparts. Similarly, Buzera et al. [12] observed higher bulk density values for oven-dried potato flour than freeze-dried potato flour. *Bonita* recorded the highest values (0.9 g/mL and 0.8 g/mL for hot-air-dried and freeze-dried flours, respectively), consistent with those reported for hot-air-dried white-fleshed SP flour [21]. *Bellevue* showed intermediate values (0.8 g/mL and 0.6 g/mL for hot-air-dried and freeze-dried flours, respectively), while *NP1648* showed the lowest bulk density (0.5 g/mL), irrespective of the drying method.

Zhang [22] stated that bulk density is closely associated with particle size. Research indicates that the HAD-induced structural collapse promotes higher particle compaction, increasing bulk density values. Conversely, FD generates a more porous structure due to water removal by sublimation, which reduces particle size and lowers bulk density values [8,12,23,24,25]. Furthermore, previous studies on sweet potato flour [21], potato flour [12], wheat and non-wheat flours [24,26], and composite flours [25] have also demonstrated that bulk density is influenced by both variety and drying method, with reported values ranging from 0.49 to 1.04 g/mL. Overall, the bulk density values observed in this study suggest that SP flours are well suited for food preparations, particularly in applications requiring blending [12].

### 2.4. Hydration and Lipophilic Properties

Table 2 shows the water (WAC) and oil (OAC) absorption capacities, swelling power (SwP), and water solubility (WS) of hot-air-dried and freeze-dried flours from the three studied varieties.

#### 2.4.1. Water and Oil Absorption Capacities

The drying method (F = 800) had a more significant influence on the water absorption capacity than the variety (F = 12). Freeze-dried flours consistently exhibited lower WAC values (≈2.6 g/g) than hot-air-dried flours (≈3.2 g/g), regardless of SP variety. The WAC values obtained in this study fall within the reported range for SP flours (1.63–7.03 g/g; [21,27,28]. A similar trend has been observed in white-fleshed SP flour [20] and quinoa flour [29], where hot-air or oven-drying yielded flours with higher WAC than freeze-dried counterparts. SEM analysis (Figure 2) revealed that FD led to the formation of aggregated particles with lamellar structures and increased overall porosity. Although higher porosity is often associated with a larger surface area, the loosely packed microstructure of freeze-dried flour may reduce capillary action, thereby limiting effective water absorption, as reported by Ahmed et al. [20]. In contrast, HAD produced a denser, more cohesive structure with fewer air pockets, potentially enhancing the accessibility of hydrophilic sites for water binding. The higher WAC observed in hot-air-dried flour has been primarily attributed to its hydrophilic components, including starch, fibres, and proteins with polar amino acid residues that readily form hydrogen bonds with water molecules [23,24,25]. Furthermore, thermal processing during HAD has been reported to induce structural modifications, such as partial starch gelatinisation or protein unfolding, which could further enhance water retention capacity [30].

Similar to WAC, the drying method (F = 845) influenced the oil absorption capacity significantly more than the variety (F = 81). Freeze-dried flours exhibited higher OAC values (≈2.9 g/g) than hot-air-dried flours (≈2.2 g/g) across all SP varieties. The values reported in this study exceeded the previously reported range for SP flours derived from differently pigmented varieties (0.95–1.07 g/g; [27]), possibly due to cultivar-specific variations. The elevated OAC in freeze-dried flours may be attributed to their highly porous and aggregated particle morphology, which provides a larger surface area and higher accessibility to voids for oil retention. In particular, the presence of lamellar structures and loosely packed starch granules appears to facilitate oil uptake. Conversely, the hot-air-dried flours exhibited a more compact and smoother morphology, with fewer internal voids and reduced surface roughness, which likely limited oil retention [31]. A similar trend was observed by Shen et al. [29], who reported that freeze-dried quinoa flour displayed significantly higher OAC than spray- and vacuum-dried counterparts, attributing this trend to an increased proportion of hydrophobic constituents. Proteins, which contain both hydrophilic and hydrophobic segments, play a key role in OAC, as their non-polar amino acid side chains can interact with the hydrocarbon chains of lipids through hydrophobic interactions [23,25]. Furthermore, Shen et al. [29] linked the higher OAC of freeze-dried quinoa flour to its greater surface hydrophobicity than spray- and vacuum-dried flours.

#### 2.4.2. Swelling Power and Water Solubility

Swelling power (SwP) exhibited significant variations primarily due to the drying method (F = 129) rather than the SP variety (F = 28). Across all varieties, hot-air-dried flours consistently displayed higher SwP than freeze-dried flours (Table 2). This difference may be attributed to the temperature-dependent nature of SwP in SP flour, as elevated processing temperatures are known to enhance starch swelling [8]. Additionally, hot-air-dried flours’ more uniform and spherical granular morphology may have enhanced water penetration and hydration, leading to a higher SwP. In contrast, the lamellar structures and partially disrupted starch granules in freeze-dried flours may have limited water absorption and restricted granule expansion during hydration, thereby reducing SwP. Other studies have reported similar explanations [32,33]. The SwP values assessed in this study align with previous reports for SP flours from different-coloured varieties, which ranged between 3.40–3.67 g/g [34] and 4.6–5.9 g/g [35]. Moreover, Kusumayanti et al. [34] observed that variety had no significant effect on SwP. Similarly, Ahmed et al. [20] reported higher SwP values for hot-air-dried SP flours (2.5 g/g) compared to freeze-dried flours (2.1 g/g), reinforcing the impact of drying conditions on SwP.

Water solubility (WS) was primarily influenced by the SP variety (F = 301.5) rather than the drying method (F = 0.2). Among the studied varieties, *Bellevue* exhibited the highest WS (≈38.3%), followed by *Bonita* (≈27.1%) and *NP1648* (≈24.2%), highlighting significant varietal differences. These findings fall within the reported WS range for SP flours from different-coloured varieties (8.6–45.7%) [27,36,37]. Previous studies have also demonstrated that varietal differences significantly affected the solubility of SP flour [36,37]. The relatively high solubility values observed in SP flour are likely attributable to its elevated soluble sugar content and amylose leaching from the starch matrix [37].

### 2.5. Foaming and Emulsifying Properties

Table 3 presents the foaming capacity and stability, as well as the emulsifying capacity and stability, of hot-air-dried and freeze-dried flours from the three studied varieties.

The drying method significantly influenced the foaming properties of SP flours (F = 113) rather than variety (F = 9). Across all varieties, freeze-dried flours exhibited superior foaming capacity (≈17.6%) and stability (≈12%) compared to hot-air-dried flours (≈8.2% and 5.4%, respectively). Regardless of the drying method or variety, foam stability decreased by 32–34% one hour after whipping. Foaming capacity and stability are primarily governed by proteins’ ability to form interfacial films, stabilising air bubbles and preventing rapid coalescence. Freeze-dried flour’s porous morphology may facilitate air entrapment, increasing its foaming capacity and stability [38]. The smaller particle size of freeze-dried flour may improve dispersibility in aqueous phases, allowing for more efficient foam formation during whipping [29]. Additionally, FD preserves protein structure and functionality by minimising heat-induced denaturation, thereby enhancing the ability of proteins to reduce surface tension and form stable air–liquid interfaces. In contrast, heat denaturation reduces protein functionality, impairing foam formation and stability [23], which may explain the lower foaming properties observed in hot-air-dried flours.

On the other hand, no significant differences were observed in the emulsifying properties across drying methods or varieties, with a mean emulsification value of 43.6%. This consistency likely arises from emulsification being predominantly governed by amphiphilic molecules, such as proteins and certain starches, which facilitate interactions between water and oil phases [39]. Furthermore, the emulsifying properties of SP flours appeared to be more dependent on their intrinsic protein and starch composition than on the drying process [38]. These findings are consistent with the reported emulsion capacities (41.5–50.4%) of various wheat and non-wheat flours [24,25,26], indicating that the SP flours under investigation exhibit a relatively strong emulsifying potential.

### 2.6. Gelatinisation Properties

Table 4 presents the gelatinisation temperature (GT) and least gelation concentration (LGC) of SP flours and commercial potato starch as a reference. SP variety exerted a more decisive influence on these parameters (F ≈ 7311 and 15.5, respectively) compared to the drying method (F ≈ 264 and 0.5, respectively), suggesting that GT and LGC are primarily dependent on the intrinsic characteristics of SP varieties rather than by drying-induced microstructural modifications.

The gelatinisation temperature of all SP flours ranged from 74 to 83 °C, exceeding the typical GT of commercial potato starch (ranging between 60 and 70 °C [40]), indicating enhanced thermal stability. Among the studied varieties, *Bonita* flours exhibited the highest GT, followed by *NP1648* and *Bellevue*. The observed varietal differences in gelatinisation properties can be attributed to intrinsic compositional variations, particularly starch content and the amylose-to-amylopectin ratio. Previous studies have shown that starch composition is key in modulating thermal stability and gelatinisation behaviour [32,41]. For *Bonita*, its higher starch content (reported as 53.3% in Pereira et al., 2024 [11]) likely contributed to the elevated gelatinisation temperature, possibly due to stronger intermolecular interactions within the starch granules. This observation is consistent with the findings of Yang et al. (2025) [41], who reported a positive correlation between amylose content and gelatinisation temperature in SP starches. In contrast, *Bellevue* and *NP1648* varieties showed lower starch contents (48.9% and 45.0%, respectively, as reported by Pereira et al., 2024 [11]), which may account for their reduced thermal stability. Regarding the influence of drying methods, *Bonita’s* hot-air-dried flour exhibited a higher GT (82.8 °C) than its freeze-dried counterpart (80.6 °C). Conversely, for *Bellevue* and *NP1648*, freeze-dried flours displayed higher GTs (75.7 °C and 81.6 °C, respectively) than hot-air-dried flours (74.3 °C and 78.5 °C, respectively). However, these method-dependent variations were below 3 °C and followed no discernable trend, suggesting that the drying method had a minor impact. Similar GT ranges for different-coloured SP flours have been reported, such as 78.5–79.8 °C [42] and 58–84 °C [43]. Desale and Sasanatayart [44] also found no significant differences in GT between freeze-dried and hot-air-dried SP flours, further supporting the minor impact of drying methods on this parameter.

Several factors influence the gelatinisation properties of flour. The intrinsic properties of starch, particularly the amylose-to-amylopectin ratio and crystallite arrangement, are likely the dominant factors determining GT [32]. These findings corroborate the lower GT observed in commercial potato starch, which is consistent with its highly purified starch content. Moreover, the absence of non-starch components, such as proteins and fibres, reduces resistance to gelatinisation, allowing starch granules to gelatinise more easily [45]. Conversely, sugars may compete with starch for water, delaying gelatinisation and increasing the final temperature required for complete gelatinisation [23].

**Figure 3:**
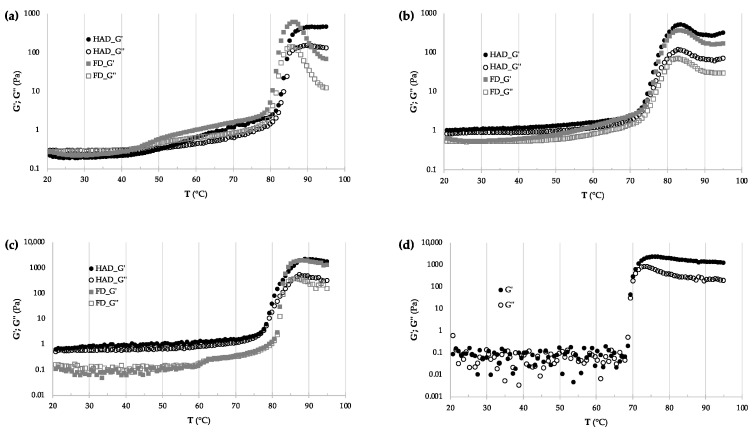
Rheological properties (storage modulus, G′, and loss modulus, G″) of hot-air-dried and freeze-dried flours from three SP varieties: (**a**) *Bonita*, (**b**) *Bellevue*, (**c**) *NP1648*, and (**d**) a commercial potato starch.

Figure 3 illustrates the distinct rheological behaviour of SP flours compared to commercial potato starch by showing the variations in storage modulus (G′) and loss modulus (G″) as a function of temperature (20–95 °C).

In commercial potato starch, the G′ and G″ values increase exponentially, whereas in SP flour, the increase is more gradual. In potato starch, which is primarily composed of amylose and amylopectin, heating promotes rapid gelatinisation and the formation of a dense, uniform network, resulting in a sharp increase in both G′ and G″. On the other hand, SP flours contain additional components, such as proteins, dietary fibres, and non-starch polysaccharides, which may interfere with the gelatinisation process by restricting starch granule swelling and hindering the development of a continuous gel matrix. Consequently, the viscoelastic properties of SP flour develop more progressively, as reflected by the slower increase in G′ and G″ values. This distinction underscores the impact of compositional complexity on those ingredients’ thermal and rheological behaviours.

Regarding the least gelation concentration, *NP1648* flours exhibited the lowest value (10%) across both drying methods, while *Bonita* and *Bellevue* flours displayed similar LGC values, ca. 16%, irrespective of the drying method. The good gelling characteristics can be attributed to carbohydrates (mainly starch) and proteins, which contribute to gel formation [23]. These findings suggest that the drying process did not significantly affect the molecular interactions responsible for gelation. Similar results were reported by Ngoma et al. [21], who noted that drying methods had no significant effect on the LGC of SP flour. Moreover, the authors found that all SP flours required a low concentration (10%, *w*/*v*) to form a gel.

### 2.7. PCA Analysis

To better visualise the interaction between the effects of the drying method and SP variety on flour’s physical and functional properties, a multivariate analysis (PCA and Hierarchical Clustering) was performed. A preliminary analysis was conducted to identify the quantitative variables that significantly contributed to the model. Variables related to gelatinisation properties, such as gelatinisation temperature and least gelation concentration, were excluded due to low factor loadings. The final data matrix comprised 10 quantitative variables and 18 samples, categorised by SP variety (*Bonita*—‘Bon’, *Bellevue*—‘Bell’, and *NP1648*—‘NP’) and drying method (HAD and FD). The selected variables (loading factors provided in Supplementary Material Table S1) included particle size, WAC, OAC, bulk density, SwP, WS, foaming capacity and stability, and emulsifying capacity and stability.

**Figure 4:**
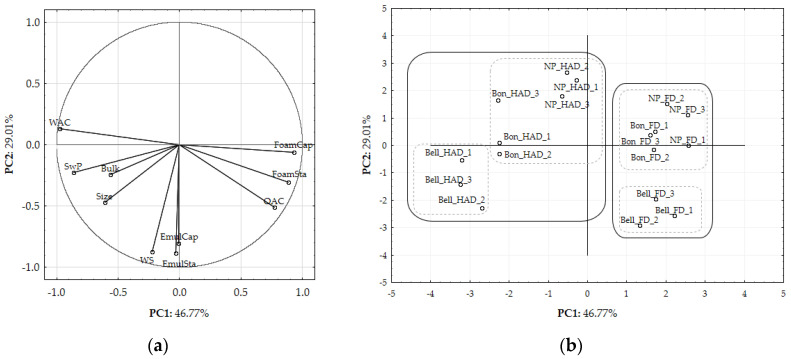
Principal component analysis (PCA) of the physical and functional properties of SP flours, influenced by variety and drying method: (**a**) loading plot and (**b**) score plot. Abbreviations: Size—particle size, WAC—water absorption capacity, OAC—oil absorption capacity, Bulk—bulk density, SwP—swelling power, WS—water solubility, FoamCap—foaming capacity, FoamSta—foaming stability, EmulCap—emulsifying capacity, EmulSta—emulsifying stability.

Figure 4 shows the spatial projection plots, illustrating the ordination of the variable vectors (a) and the distribution of the samples (b) in the plane defined by the first two principal components. The PCA identified two principal components—PC1 and PC2—that accounted for 75.78% of the total variance in the dataset.

The first principal component (PC1) accounted for 46.77% of the total variance, with the highest loadings observed for particle size, water absorption capacity (WAC), oil absorption capacity (OAC), swelling power (SwP), and foaming properties (both capacity and stability). Specifically, OAC and foaming properties correlated positively with PC1, whereas WAC, SwP, and particle size showed negative correlations. These results suggest that PC1 is predominantly associated with the flour’s microstructure, which depends on the drying method (as discussed in Section 2.2). Notably, the inverse relationship between OAC and WAC indicates that the flours exhibit a preferential affinity, predominantly hydrophilic or lipophilic. The second principal component (PC2) explained 29.01% of the total variance and was primarily driven by water solubility (WS) and emulsifying properties, all correlated negatively with PC2. The variables defining PC2 appear to be independent of the drying method, with WS in particular being influenced by the specific variety under study (as discussed in Section 2.4). The distribution of the samples along PC1 indicated that those positioned on the left side exhibited higher WAC, SwP, bulk density, and particle size, while samples on the far right displayed greater OAC and foaming ability. The positioning of the samples along the lower axis of PC2 was associated with increased WS and enhanced emulsifying properties.

The score plot (Figure 4b) revealed sample groupings consistent with the hierarchical cluster dendrogram (Supplementary Material Figure S1). The first division in the hierarchical clustering (linkage distance < 6) separated hot-air-dried flours from freeze-dried flours, independent of SP variety. The positioning of freeze-dried flours on the far right of PC1 indicated a strong association with functional properties such as OAC, foaming capacity, and foaming stability. In contrast, hot-air-dried flours were positioned on the left side of PC1, closely associated with higher WAC, SwP, and bulk density. The subsequent cluster (linkage distance < 5) further differentiated the *Bellevue* flours from the other varieties, associating them with higher water solubility and emulsifying properties. The sample grouping corroborates what was mentioned earlier regarding the effects of the drying method and SP variety. Overall, the observed differentiation highlights the significant role of drying method rather than variety in modulating the functional properties of SP flours.

Interestingly, while the physical and functional properties of SP flours were predominantly influenced by the drying method, the retention of bioactive compounds in SP flours was more dependent on variety, particularly in preserving key phytochemicals such as carotenoids and phenolics [11].

## 3. Materials and Methods

### 3.1. Plant Material and Sweet Potato Flour Preparation

**Figure 5:**
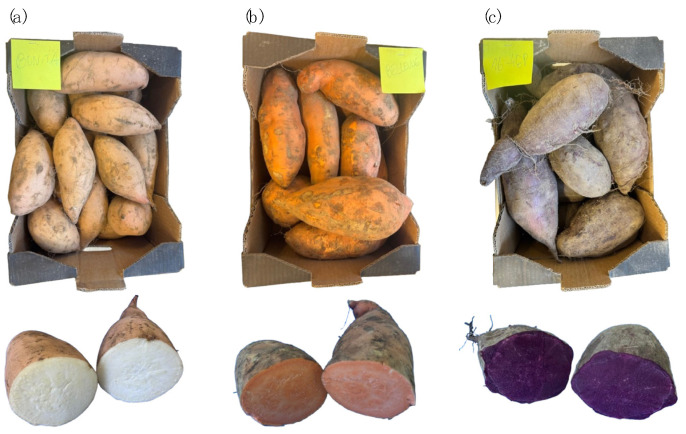
Varieties under study: (**a**) *Bonita*, white-fleshed; (**b**) *Bellevue*, orange-fleshed; (**c**) *NP1648*, purple-fleshed.

The SP cultivars were produced in Portugal by NativaLand, Lda., and were selected for their phenotypic and compositional diversity and adaptability to national edaphoclimatic conditions. The study focused on three varieties: *Bonita* (white-fleshed), *Bellevue* (orange-fleshed), and *NP1648* (purple-fleshed) (Figure 5). The tubers were harvested in 2023 and subsequently subjected to a controlled curing process at 30 °C and 95% relative humidity, with adequate ventilation for 2–7 days. Thereafter, the tubers were stored under regulated conditions (12–15 °C; 80% relative humidity; adequate ventilation) for up to one week before being transported to the INIAV laboratory for further analysis.

This study implemented a 2 × 3 full factorial design with two independent variables: drying method (hot-air and freeze-drying) and SP variety (*Bonita*, *Bellevue*, and *NP1648*). This approach yielded six distinct experimental combinations and 18 samples, enabling the assessment of both main effects and interactions between factors.

Flour production entailed drying and milling processes and was conducted at the TagusValley—Science and Technology Park (Abrantes, Portugal). Initially, all SP varieties were cut into 4 mm thick slices and then subjected to two drying methods, as described by Pereira et al. [11]: hot-air drying (HAD) at 75 °C for 20 h (Klarstein Master Jerky 32, Berlin, Germany) and freeze-drying (FD) at a temperature range of −41 to 30 °C for 70 h (Coolvacuum Lyobiotic 10FD, Barcelona, Spain). The dried samples were subsequently milled into flour using a food processor (Vorwerk, Wuppertal, Germany) at maximum speed for 1 min, sieved through a 250 µm mesh, vacuum-packed in LPDE films and stored at −80 °C until further analysis. The flours were characterised according to the procedures detailed in Section 3.2.

### 3.2. Methods for Assessing the Physical and Functional Properties of Flours

#### 3.2.1. Particle Size Distribution

A laser scattering particle size distribution analyser (Partica LA-960, Horiba Scientific, Amadora, Portugal), equipped with a dry feeder accessory, was used to measure the particle size distribution of flours. During measurement, the flours were placed into the hopper with a 3 mm hopper gap and fed into the detection unit through airflow, using settings of 2 bar pressure and a 100% feed rate via a vibrational feeder. The particle size (µm) distribution was determined based on volume, with each flour analysed in triplicate.

#### 3.2.2. Morphology of the Starch Granules

The morphology of the native starch granules was evaluated using a scanning electron microscope equipped with a secondary electron detector (Phenon Prox G6, Thermo Scientific). Flours were mounted on the sample stage with conductive double-sided adhesive tape, and a thin layer of gold–palladium was sputtered onto the sample surface. Images were captured at magnifications 300×, 700×, 1500×, and 3000× under an accelerating voltage of 10 kV.

#### 3.2.3. Bulk Density

Bulk density was measured following the method of Kaur and Singh [46]. About 10 g of SP flour was placed in a 50 mL graduated cylinder and tapped gently until tightly packed. Bulk density was calculated as the flour’s weight (g) ratio to its packed volume (mL).

#### 3.2.4. Hydration and Lipophilic Properties

The water absorption capacity (WAC) and oil absorption capacity (OAC) of SP flours were measured according to Chikpah et al. [47]. Approximately 0.5 g of flour (W1) was placed into a pre-weighted centrifuge tube (W2) and dispersed in distilled water (5 mL for WAC) or palm olein oil (5 mL for OAC). The mixtures were vortexed (1 min; Heidolph REAX 2000, Schwabach, Germany), allowed to stand for 30 min at room temperature, and then centrifuged (4000 rpm/30 min; Sigma 2k15 Laborzentrifugen, Osterode am Harz, Germany). The pellet was weighted (W3), and the WAC and OAC were calculated using the following equation and expressed in grams of water or oil absorbed per gram of flour:

$$WAC/OAC\left(g/g\right)=\frac{W3-(W2+W1)}{W1}$$

Water solubility (WS) and swelling power (SwP) of the flours were determined as described by Chikpah et al. [47]. A pre-weighed centrifuge tube (W1) containing 1 g of flour and 12.5 mL of distilled water was heated in a water bath (Unitronic Reciprocating Shaking Bath, J.P. Selecta, Barcelona, Spain) at 60 °C for 30 min, with constant stirring (70 U/min). The slurry was centrifuged (4000 rpm/30 min; Sigma 2k15 Laborzentrifugen, Osterode am Harz, Germany), and the supernatant was transferred into a pre-weighed evaporating dish (W2) and dried at 105 °C (Memmert Incubator, Schwabach, Germany) to a constant weight (W3). The WS was calculated as follows:

$$WS\left(\%\right)=\frac{\left(W3-W2\right)}{Initial\;weight\;sample}\times 100$$

The tube containing the pellet was weighted (W4), and the SwP was calculated using the equation:

$$SwP\left(g/g\right)=\frac{\left(W4-W1\right)}{Initial\;weight\;sample}$$

#### 3.2.5. Foaming Properties

Flour foaming capacity was determined according to Chandra and Samsher [24]. About 1 g of flour was mixed with 50 mL of distilled water in a 100 mL measuring cylinder and shaken for 1 min until foam was formed, and the volume increase was recorded. The foaming capacity was expressed as the percentage increase in volume using the following equation:

$$Foaming\;Capacity\left(\%\right)=\frac{V2-V1}{V1}\times 100,$$

V_1_ and V_2_ correspond to volumes (mL) before and after whipping, respectively.

Foam stability was assessed by comparing the initial foam volume with the volume after 1 h.

#### 3.2.6. Emulsifying Properties

The emulsifying capacity was determined according to Chandra and Samsher [24]. Approximately 1 g of flour, 10 mL of distilled water, and 10 mL of palm olein oil were mixed and centrifuged (2100× *g*/5 min.). The emulsifying capacity was calculated as the ratio of the emulsion layer height to the total mixture height and expressed as a percentage using the following equation:

$$Emulsifying\;Capacity\left(\%\right)=\frac{Height\;of\;emulsion\;layer}{Height\;of\;the\;whole\;layer}\times 100$$

The emulsifying stability was assessed by heating (80 °C/30 min.) the previously prepared samples, cooling, and centrifuging (1100× *g*/5 min.) them. Results are expressed as the percentage of emulsifying capacity retained after heating.

#### 3.2.7. Gelatinisation Properties

The gelatinisation temperature of flours was measured using a controlled-stress rheometer (Haake MARS III, Thermo Scientific, Waltham, MA, USA), according to Mironeasa and Mironeasa [48], with slight modifications. A preliminary stress sweep was performed to identify the limits of the linear viscoelastic region under the following conditions: corrugated plate diameter 20 cm, plate gap 1 mm, scanning frequency 1 Hz, stress range 0.1–5000 Pa, and temperature 20 °C. A 15% suspension of each flour was prepared in distilled water, stirred for 1 h at room temperature, and then placed on the rheometer test bench. The excess sample was removed, and liquid paraffin was applied to the exposed edge to prevent moisture loss during testing. A temperature sweep was conducted to measure the storage modulus (G′) and loss modulus (G″) over a temperature ramp from 20 to 95 °C, with a heating rate of 2 °C/min, at a constant frequency of 1 Hz and an applied stress of 1 Pa. Finally, a frequency sweep was performed at 20 °C, with a frequency range of 0.1–100 Hz and an applied stress of 2 Pa.

The least gelation concentration (LGC) of SP flours was determined according to Kaur and Singh [46]. Sample suspensions of 2–30% (*w*/*v*) of each flour were prepared in 5 mL of distilled water and transferred into test tubes. The suspensions were heated for 1 h in boiling water (95 °C) (Unitronic Reciprocating Shaking Bath, J.P. Selecta, Barcelona, Spain) and then cooled under cold running water. The tubes were subsequently placed at 4 °C for 1 h. The LGC was determined as the minimum concentration at which the sample in an inverted tube did not slip or fall.

### 3.3. Statistical Analysis

Data were analysed using a two-factor factorial ANOVA in Statistica^TM^ v8.0 (2007) software from StatSoft Inc. (Tulsa, OK, USA) [49], with Tukey’s HSD test applied to identify statistically significant differences (*p* < 0.05) between samples. The F-value was used to assess the individual impact of each factor (SP variety and drying method) and their interaction with the dependent variables. A higher F-value indicates a greater contribution of that effect to the variability in the dependent variable, suggesting a stronger influence of the factors being evaluated. A principal component analysis (PCA) was also conducted. Before analysis, all variables were mean-centred and standardised (scaled) to unit variance (correlation matrix). The eigenvalues and eigenvectors of the study data’s correlation matrix were computed to derive the principal components [50]. Each sample’s score was calculated as a linear combination of each PCA component, with the parameter loadings revealing the contribution of each variable. A two-dimensional representation of this multi-dimensional dataset was generated using the primary components that accounted for over 70% of the total variance—a threshold considered sufficient to establish a robust qualitative model [51]. Furthermore, a hierarchical cluster analysis was performed; the clustering process involved data standardisation, sample dissimilarity metric assessment using Euclidean distance, and sample grouping via Ward’s method.

## 4. Conclusions

The results of this study highlight the significant influence of drying methods and sweet potato varieties on the physical and functional properties of the produced flours. The drying method affected both particle size and microstructure. Freeze-drying resulted in flours predominantly composed of small particles (≤50 µm), characterised by a porous microstructure and partially fragmented starch granules. In contrast, hot-air drying produced flours with two distinct particle size classes (≤50 µm and >200 µm) and a more compact microstructure, better preserving the starch granules’ integrity. These differences impacted the physical and functional properties, with freeze-dried flours exhibiting greater oil affinity and hot-air-dried flours demonstrating higher water absorption and compaction indices. Furthermore, the sweet potato variety primarily influenced water solubility and gelatinisation properties, determined by the intrinsic chemical composition—particularly the starch characteristics (e.g., amylose/amylopectin ratio) and the presence of non-starchy compounds such as proteins, fibres, and reducing sugars.

Additionally, all SP flours displayed high emulsifying capacity values (≈44%), underscoring their potential in applications such as salad dressings, yoghurts, and other food formulations. Based on the evaluated properties, freeze-dried flours, with their enhanced oil absorption and foaming capabilities, are well suited for aerated and light-textured products like cakes, muffins, and mousse-based desserts, as well as for snacks where oil retention contributes to crispiness. Conversely, hot-air-dried flours, characterised by higher water absorption, swelling power, and bulk density, are recommended for formulations that require moisture retention and structural integrity, such as dense bread, pancakes, porridges, soups, stews, gluten-free pasta, and energy bars. This study provides valuable insights into the interplay between drying methods and variety-specific characteristics, establishing a solid foundation for tailoring sweet potato flour processing for diverse food applications.

## Figures and Tables

**Table 1:** Average values (±SD) of bulk density (g/mL) for hot-air-dried and freeze-dried flours from three SP varieties (*Bonita*, *Bellevue*, and *NP1648*). Different letters indicate significant differences (*p* < 0.05) for the bulk density column.

Variety	Drying Method	Bulk Density (g/mL)
*Bonita*	HAD	0.9 ^d^ ± 0.1
	FD	0.8 ^c^ ± 0.1
*Bellevue*	HAD	0.8 ^c^ ± 0.1
	FD	0.6 ^b^ ± 0.1
*NP1648*	HAD	0.5 ^a^ ± 0.1
	FD	0.5 ^a^ ± 0.1

**Table 2:** Average values (±SD) of water absorption capacity (WAC, g water absorbed/g flour), oil absorption capacity (OAC, g oil absorbed/g flour), swelling power (g/g), and water solubility (%) for hot-air-dried and freeze-dried flours from three SP varieties (*Bonita*, *Bellevue*, and *NP1648*). For each column, different letters indicate significant differences (*p* < 0.05).

Variety	Drying Method	WAC (g/g)	OAC (g/g)	SwP (g/g)	WS (%)
*Bonita*	HAD	3.2 ^b^ ± 0.1	2.2 ^a^ ± 0.1	3.2 ^d^ ± 0.1	27.8 ^b^ ± 0.9
	FD	2.6 ^a^ ± 0.0	2.5 ^b^ ± 0.1	2.7 ^a^ ± 0.1	26.4 ^ab^ ± 0.5
*Bellevue*	HAD	3.2 ^b^ ± 0.1	2.2 ^a^ ± 0.1	3.6 ^e^ ± 0.1	37.0 ^c^ ± 0.5
	FD	2.6 ^a^ ± 0.0	3.4 ^d^ ± 0.1	3.0 ^bc^ ± 0.1	39.5 ^c^ ± 1.2
*NP1648*	HAD	3.0 ^c^ ± 0.0	2.2 ^a^ ± 0.0	3.1 ^cd^ ± 0.1	24.4 ^a^ ± 2.0
	FD	2.6 ^a^ ± 0.1	2.9 ^c^ ± 0.1	2.9 ^ab^ ± 0.1	23.9 ^a^ ± 0.3

**Table 3:** Average values (±SD) of foaming capacity (%), foaming stability (%), emulsifying capacity (%) and emulsifying stability (%) for hot-air-dried and freeze-dried flours from three SP varieties (*Bonita*, *Bellevue*, and *NP1648*). For each column, different letters indicate significant differences (*p* < 0.05).

Variety	Drying Method	Foaming Capacity (%)	Foaming Stability (%)	Emulsifying Capacity (%)	Emulsifying Stability (%)
*Bonita*	HAD	6.1 ^a^ ± 0.2	4.0 ^a^ ± 0.2	44.3 ^a^ ± 0.9	43.3 ^ab^ ± 1.5
	FD	16.6 ^b^ ± 0.2	12.4 ^b^ ± 0.2	44.3 ^a^ ± 0.4	42.8 ^ab^ ± 0.4
*Bellevue*	HAD	8.3 ^a^ ± 0.4	6.3 ^a^ ± 0.4	44.6 ^a^ ± 0.9	43.6 ^ab^ ± 0.9
	FD	16.1 ^b^ ± 0.8	11.8 ^b^ ± 1.6	45.1 ^a^ ± 1.1	44.6 ^a^ ± 0.4
*NP1648*	HAD	10.1 ^a^ ± 0.1	6.0 ^a^ ± 0.1	43.3 ^a^ ± 0.8	41.4 ^b^ ± 0.3
	FD	20.1 ^b^ ± 0.9	11.8 ^b^ ± 1.4	43.8 ^a^ ± 0.5	42.4 ^ab^ ± 1.5

**Table 4:** Average values (±SD) of gelatinisation temperature (GT; °C) and least gelation concentration (LGC; %) for hot-air-dried and freeze-dried flours from three SP varieties (*Bonita*, *Bellevue*, and *NP1648*) and commercial potato starch. For each column, different letters indicate significant differences (*p* < 0.05).

Variety	Drying Method	GT (°C)	LGC (%)
*Bonita*	HAD	82.8 ^f^ ± 0.1	16 ^a^ ± 2
	FD	80.6 ^d^ ± 0.1	16 ^a^ ± 2
*Bellevue*	HAD	74.3 ^a^ ± 0.1	14 ^ab^ ± 2
	FD	75.7 ^b^ ± 0.1	16 ^a^ ± 2
*NP1648*	HAD	78.5 ^c^ ± 0.1	10 ^b^ ± 2
	FD	81.6 ^e^ ± 0.1	10 ^b^ ± 2
Commercial potato starch	HAD	68.7 ^g^ ± 0.1	-

## Data Availability

Data will be made available to the corresponding author upon reasonable request.

## Supplementary Materials

The following supporting information can be downloaded at https://www.mdpi.com/article/10.3390/molecules30081846/s1, Table S1: Factor loading on the two principal components of each variable; Figure S1: Hierarchical cluster analysis dendrogram of the data matrix.
