# Supplementary material for: Physical and Functional Properties of Sweet Potato Flour: Influence of Variety and Drying Method
Source: Molecules. 2025 Apr 20;30(8):1846. doi: 10.3390/molecules30081846 (PMC12029891; doi:10.3390/molecules30081846)
Supplement: Supplementary file 1 [file molecules-30-01846-s001.zip › molecules-3569185-supplementary.pdf]

Article

Physical and Functional Properties of Sweet Potato Flour: Influence of Variety and Drying Method

Nelson Pereira [1,2], Ana Cristina Ramos [1,3], Marco Alves [4], Vítor D. Alves [2,5], Margarida Moldão [2,5], Marta Abreu [1,2,5*]

1 INIAV - Instituto Nacional de Investigação Agrária e Veterinária, Unidade de Tecnologia e Inovação, Oeiras, Portugal; isa128286@isa.ulisboa.pt (N.P); cristina.ramos@iniav.pt (A.C.R); cristina.roseiro@iniav.pt (C.R); manuela.vida@iniav.pt (M.V); marta.abreu@iniav.pt (M.Ab)
2 LEAF - Linking Landscape, Environment, Agriculture and Food Research Center, Instituto Superior de Agronomia, ULisboa, Lisboa, Portugal; vitoralves@isa.utl.pt (V.D.A); mmoldao@isa.utl.pt (M. M)
3 GeoBioTec - Geobiociências, Geoengenharias e Geotecnologias, FCT-UNL, Caparica, Portugal
4 INOV.LINEA/TAGUSVALLEY – Science and Technology Park, Abrantes, Portugal; marco_alves@tagusvalley.pt (M.Al)
5 Associate Laboratory TERRA, Instituto Superior de Agronomia, ULisboa, Lisboa, Portugal
* Correspondence: marta.abreu@iniav.pt (M.Ab)

Supplementary Material

Tables

Supplementary Material Table S1 | Factor loading on the two principal components of each variable

Variable	PC1	PC2
Size	-0.61	-0.47
WAC	-0.97*	0.13
OAC	0.77*	-0.51
Bulk	-0.56	-0.24
Swelling	-0.86*	-0.23
WaterSolub	-0.22	-0.87*
FoamCap	0.93*	-0.06
FoamSta	0.89*	-0.31
EmulCap	-0.00	-0.81*
EmulSta	-0.03	-0.88*

* marked loading are > 0.70.

Figures

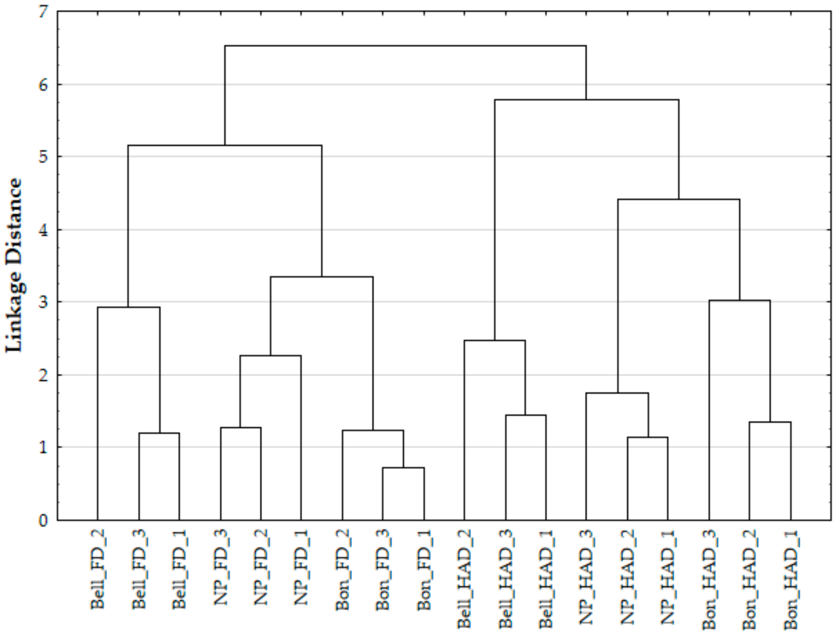

Supplementary Material Figure S1 | Hierarchical cluster analysis dendrogram of the data matrix.
